# Gene co-expression networks are associated with obesity-related traits in kidney transplant recipients

**DOI:** 10.1186/s12920-020-0702-5

**Published:** 2020-03-10

**Authors:** Rosario B. Jaime-Lara, Abhrarup Roy, Yupeng Wang, Ansley Stanfill, Ann K. Cashion, Paule V. Joseph

**Affiliations:** 1 Division of Intramural Research, National Institute of Nursing Research, National Institutes of Health, Bethesda, MD 20892 USA; 2 BDX Research & Consulting LLC, Herndon, VA 20171 USA; 3 The University of Tennessee Health Science Center, College of Nursing, Memphis, Memphis, TN 38163 USA

**Keywords:** Co-expression modules, Obesity, Association, Enrichment analysis, Gene ontology, Kidney transplant

## Abstract

**Background:**

Obesity is common among kidney transplant recipients; However biological mediators of obesity are not well understood in this population. Because subcutaneous adipose tissue can be easily obtained during kidney transplant surgery, it provides a unique avenue for studying the mechanisms of obesity for this group. Although differential gene expression patterns were previously profiled for kidney transplant patients, gene co-expression patterns can shed light on gene modules not yet explored on the coordinative behaviors of gene transcription in biological and disease processes from a systems perspective.

**Methods:**

In this study, we collected 29 demographic and clinical variables and matching microarray expression data for 26 kidney transplant patients. We conducted Weighted Gene Correlation Network Analysis (WGCNA) for 5758 genes with the highest average expression levels and related gene co-expression to clinical traits.

**Results:**

A total of 35 co-expression modules were detected, two of which showed associations with obesity-related traits, mainly at baseline. Gene Ontology (GO) enrichment was found for these two clinical trait-associated modules. One module consisting of 129 genes was enriched for a variety of processes, including cellular homeostasis and immune responses. The other module consisting of 36 genes was enriched for tissue development processes.

**Conclusions:**

Our study generated gene co-expression modules associated with obesity-related traits in kidney transplant patients and provided new insights regarding the cellular biological processes underlying obesity in this population.

## Background

Obesity is a growing health concern worldwide and is associated with renal co-morbidities such as Chronic Kidney Disease (CKD), End Stage Renal Disease (ESRD), and other kidney complications [1–7]. In 2011, 23% of kidney transplant recipients in the United States were obese, 9.4% were morbidly obese, and 2.1% were morbidly obese [8]. In addition, post-transplantation weight gain in kidney transplant cohorts is common, thus exacerbating patients’ preexisting obese phenotype and decreasing the likelihood of long-term renal allograft success [9, 10].

Obesity is influenced by the interaction of genetic and environmental factors [11–13], and as obesity prevalence increases and genetic methods evolve, there is a growing interest in studying the genetic and biological mechanisms driving weight gain. Several studies have investigated genetic mechanisms of obesity through various methodologies, including gene expression studies, Genome Wide Association Studies (GWAS), and obesity-related biomarkers [14, 15]. However, more comprehensive systems-based genetic analyses, which could provide a more robust understanding of gene interactions, pathways, and biological functions, are underexplored. Such inquiry could provide a more robust understanding of gene interaction networks, their pathways, and biological functions. Weighted gene correlation network analysis (WGCNA) is one such systems-based approach that offers the ability to create modules of gene networks (groups of co-expressed genes) that are highly associated with clinical variables of interest (e.g., Body Mass Index).

In addition to underutilizing systems-based approaches to study obesity, very few researchers have conducted genetic studies in human subcutaneous adipose tissue. Collecting human tissue, including adipose tissue, involves invasive procedures, making other noninvasive methods of sample collection (i.e., blood, stool, and saliva) more feasible and attractive to research participants. However, subcutaneous adipose tissue can be easily obtained during kidney transplant surgery. Furthermore, subcutaneous adipose tissue is a particularly promising candidate for gene studies of obesity, because it is metabolically active and plays an important role in endocrine pathways that modulate eating behavior and metabolism (e.g., appetite regulation, insulin signaling, and leptin signaling) [16].

A recent study by Joseph at al. carried out an analysis of gene co-expression patterns associated with body mass index using expression data in whole blood cells [17]. However, it is still unknown how gene co-expression patterns in adipose tissue are related to obesity. A longitudinal study by Cashion et al. analyzed gene expression data from a cohort of kidney transplant recipients to identify individual genes and molecular pathways that could be driving weight gain [18]. Differential gene expression analysis revealed that changes in gene expression were associated with insulin, inflammatory signaling pathways, and leptin. Their analysis also identified four obesity-associated genes (*CPE*, *LEP*, *NPY1R*, and *NPY5R*) that were positively correlated with weight gain and two genes (*APOM* and *CRP*) that were negatively correlated with weight gain. Cashion et al. also found demographic and environmental factors were associated with weight gain following kidney transplantation [19]. Although Cashion et al. compared expressed genes between those who did and did not gain weight, they did not analyze the interaction patterns among expressed genes. The current study builds on these previous findings by Cashion et al. and utilizes gene expression data to analyze gene co-expression networks to examine obesity-related traits in kidney transplant recipients [18].

## Methods

### Materials and methods

#### Design and setting

This study utilized gene expression data to examine gene interactions in human adipose tissue of kidney transplant recipients. Tissue samples were obtained from investigators at a regional MidSouth transplant center, who recruited 153 transplant recipients from 2006 to 2011 to study the genetic and environmental factors associated with weight gain following kidney transplantation. Written informed consent was obtained from all participants, and some patients also signed an optional repository consent. The parent study was approved by the Institutional Review Board of the University of Tennessee Health Science Center. Additional analyses on the repository samples were approved by the Office of Human Subjects Research and the Institutional Review Board of the National Institutes of Health.

All adults, regardless of race or sex, were eligible for participation. To control for the effect of pre-transplantation immunosuppressant therapy on gene expression profiles, exclusion criteria included prior treatment with prednisone or other immunosuppressant therapies. Other exclusion criteria included underweight status (i.e., BMI < 18.5 kg/m^2^) and pre-existing conditions that may impact weight, including gastrointestinal, pulmonary, neurologic, or gynecological diseases. Of the 153 transplant recipients, thirty signed a repository consent and also had RNA microarray data. Of these thirty participants, three were excluded due to excessive weight loss, which was determined to be a potential indicator of an abnormal recovery. An additional participants’ sample was excluded as it did not meet quality control criteria. A total of 26 participant samples were included in the current study.

#### Demographic and clinical variables

Demographic data (age, race, and sex) were collected from electronic medical records (Table 1). Clinical laboratory values were collected at the time of transplantation and at 3, 6, and 12 months post transplantation. Clinical variables were analyzed including height, weight, BMI, body fat, creatinine levels, and glucose levels. Body weight and height were used to calculate BMI (weight in kilograms divided by height in meters squared). Weight categories were selected as recommended by the Centers for Disease Control, with greater than 30 kg/m^2^ indicating obesity. Body fat was determined using dual-energy X-ray absorptiometry (DEXA) scans. Blood samples were collected to determine laboratory values (e.g., fasting glucose and creatinine). Reference values of normality for creatinine and fasting glucose levels were 0.5–1.2 mg/dL and 70–100 mg/dL respectively.

**Table 1:** Demographic characteristics of the study cohort (*N* = 26)

Characteristic	Value
Age at transplantation, mean (range)	47.7 (19~67)
Sex, n (%)	
Male	11 (42.3%)
Female	15 (57.7%)
Race, n (%)	
African American	15 (57.7%)
Caucasian	11 (42.3%)

The baseline clinical variables included weight (WT_BL), height, total subcutaneous body fat (SubTot_Fat_BL), percent of subcutaneous body fat (SubTot_Pfat_BL), whole-body fat percent (WB_Tot_Pfat_BL), body mass index (BMI_BL), blood creatinine (CREATININE_BL), and blood glucose (GLUCOSE_BL). The following variables were also measured at 3, 6, and 12 months after kidney transplantation: blood creatinine (CREATININE_3M, CREATININE_6M, CREATININE_12M) and blood glucose (GLUCOSE_3M, GLUCOSE_6M, GLUCOSE_12M). Other variables were measured at 12 months, including SubTot_Fat_12M, SubTot_Pfat_12M, and BMI_12M.

Additionally, other pre-existing conditions (i.e. hyperlipidemia, hypertension, diabetes and depression) were also collected from the medical charts and controlled for in our analysis. Adipose tissue samples were collected for all eligible participants at the time of transplant surgery. Participants followed similar immunosuppressant therapy for 6 months following transplantation, 80% received 20 mg prednisone, 8% received 10 mg of prednisone, and 4% received 50 mg of prednisone. Six months after undergoing a kidney transplant, 92% received 5 mg of prednisone and 8% did not take prednisone.

#### Microarray data processing and annotation

As a part of the parent study, RNA was extracted from adipose tissue samples and gene expression values were obtained by the authors and Cashion et al. [18]. For RNA extraction procedures please refer to Cashion et al. [18]. The expression values were generated by Affymetrix Human Gene 1.0 ST Array (GEO platform ID: GPL6244) and results were made available via GEO (https://www.ncbi.nlm.nih.gov/geo/, Dataset ID: GSE33070; annotation file: GPL6244.annot). These microarray data are publicly available, although due to privacy concerns only limited amount of demographic and clinical data for these subjects are publicly available. Such data were available to the authors on this paper through this current collaboration, though they are still limited to the repository dataset (specific available variables are described above).

The microarray data were annotated using custom programs to process the annotation file and to generate mapping between genes and transcript IDs. For each gene, we selected the transcript with the highest average expression level to represent the expression of that gene. Using this process, we obtained a total of 19,192 gene expression profiles.

### Constructing gene co-expression networks

A total of 5758 (30%) highly expressed genes were selected for co-expression network analysis. The WGCNA package was used to construct gene co-expression networks and examine their associations with clinical variables [20]. A soft-threshold power of 7 was used as it met scale-free topology criteria (*R*^*2*^ *≈ 0.8)* while generating reasonable module sizes (mean = 37) (Fig. S1). Then, one-step network construction and module detection were performed with the following parameters: TOMType = “unsigned”, minModuleSize = 10, reassign Threshold = 0, and mergeCutHeight = 0.25. Next, for each detected gene module, its eigenvector (vectors associated with linear system equations) was computed. Module-trait associations were assessed based on the Pearson’s correlation between the eigenvector of each module and each clinical variable. Original *p*-values of module-trait associations were adjusted for multiple testing using the Benjamini-Hochberg (i.e. False Discovery Rate or FDR) approach [21].

### Enrichment analysis

Functional enrichment analysis was performed using the online version of GOStat (http://gostat.wehi.edu.au/cgi-bin/goStat.pl) [22]. Parameters were chosen as follows- database: goa_human; minimal length of considered GO paths: 3; maximal *p*-value: 0.05; maximum number of GOs: 30; cluster GOs: 5; direction: over-represented; correct for multiple testing: false discovery rate. The 5758 highly expressed genes were used as a genomic background to examine enrichment of co-expression gene modules.

### Identification of hub genes in co-expression gene modules

Hub genes were identified using topology similarity as described previously by Joseph et al. [17]. Briefly, we used topology similarity to measure the comparability between gene expression profiles, and computed the topological matrix for the 5758 highly expressed genes. Subsequently, 95% quantile of the topological matrix was used as the cutoff for determining whether two genes were connected. The connectivity of a gene was the number of genes connected with it in the same gene module. This hub gene identification method was custom built using R software. In our analysis, hub genes in co-expression gene modules were defined as genes with high connectivity (ranked at the top 10%) in the candidate modules.

### Pathway analysis

Ingenuity pathway analysis (IPA), https://www.qiagenbioinformatics.com/products/ingenuity-pathway-analysis/ was used for functional analysis, integration, and interpretation of the biological role of the magenta and darkgreen modules. One hundred twenty-nine genes within the magenta module and 36 genes within the darkgreen module were input separately into IPA and a “Core Analysis” was used to construct canonical pathways and networks. Canonical pathways were constructed to examine the role of genes within each module (i.e. their role in cell signaling and metabolic pathways). Networks were constructed to identify and visualize gene-gene interactions, each connection representing known relationships between genes. Identified networks were scored based on the degree of relevance (i.e. -log (Fisher’s Exact test)) of their genes with a list of biological functions stored in the Ingenuity Knowledge Base. The Human Gene 1.0 ST Array was used as the reference background in order to reduce bias towards pathways identified in the default of option which uses the entire “Ingenuity Knowledge Base (Genes Only)” as a reference set. Additionally we ran the 5758 highly expressed genes separately and compared the results. We report the findings, canonical pathways and networks, for the module results.

## Results

### Study data

The demographic characteristics of our 26 kidney transplant recipients are summarized in Table 1. The clinical characteristics for these same recipients are shown in Table 2. A total of 26 different clinical variables were measured.

**Table 2:** Clinical characteristics of the study cohort (N = 26)

Clinical parameter	Mean ± Standard deviation	Range (min; max)
Weight at Baseline		
WT_BL (lbs)	174.80 ± 37.54	129.19; 257.90
Weight at 3 Months		
WT_3M (lbs)	170.22 ± 38.79	123.00; 242.00
Weight at 6 Months		
WT_6M (lbs)	179.96 ± 39.91	133.00; 259.80
Weight at 12 Months		
WT_12M (lbs)	182.69 ± 47.33	128.00; 294.62
BMI at Baseline		
BMI_BL (kg/m^2^)	27.41 ± 3.36	22.30; 33.62
BMI at 3 Months		
BMI_3M (kg/m^2^)	26.68 ± 3.82	20.52; 34.85
BMI at 6 Months		
BMI_6M (kg/m^2^)	28.21 ± 3.73	22.30; 34.70
BMI at 12 Months		
BMI_12M (kg/m^2^)	28.58 ± 4.97	22.64; 39.95
Weight Change at 3 Months		
WT_CHG_3M (%)	−2.71 ± 6.10	−13.92; 8.13
Weight Change at 6 Months		
WT_CHG_6M (%)	3.01 ± 6.69	−12.02; 14.24
Weight Change at 12 Months		
WT_CHG_12M (%)	4.11 ± 10.83	−17.00; 25.84
Subtotal Body Fat at Baseline		
SubTot_Fat_BL (g- below the head)	22,006.51 ± 7037.55	7505.83; 35,429.71
Percent Body Fat at Baseline		
SubTot_Pfat_BL (%)	29.79 ± 7.39	14.05; 38.47
Whole Body Fat at Baseline		
WB_Tot_Pfat_BL (g)	29.25 ± 6.95	14.50; 37.60
Subtotal Body Fat at 12 Months		
SubTot_Fat_12M (g- below the head)	26,416.99 ± 9604.71	14,528.39; 49,349.50
Percent Body Fat at 12 Months		
SubTot_Pfat_12M (%)	34.07 ± 6.85	19.20; 50.19
Whole Body Fat at 12 Months		
WB_Tot_Pfat_12M (%)	33.28 ± 6.52	19.25; 48.84
Creatininie at Baseline		
CREATININE_BL (mg/dL)	6.24 ± 3.60	1.20; 14.10
Glucose at Baseline		
GLUCOSE_BL (mg/dL)	116.50 ± 44.23	70.00; 234.00
Creatinine at 3 Months		
CREATININE_3M (mg/dL)	1.58 ± 0.42	1.00; 2.50
Glucose at 3 months		
GLUCOSE_3M (mg/dL)	125.68 ± 51.46	70.00; 266.00
Creatininte at 6 Months		
CREATININE_6M (mg/dL)	1.42 ± 0.28	1.00; 1.90
Glucose at 6 Months		
GLUCOSE_6M (mg/dL)	121.48 ± 37.72	79.00; 224.00
Creatinine at 12 Months		
CREATININE_12M (mg/dL)	1.60 ± 0.45	0.85; 2.68
Glucose at 12 Months		
GLUCOSE_12M (mg/dL)	135.29 ± 59.50	78.00; 285.00
Height (in)	66.61 ± 4.07	60.00; 75.00

The adipose tissue samples were used to generate raw RNA expression data as a part of a previous study [18]. These samples were taken at the time of transplantation surgery, and thus expression data were available for each subject at baseline. We further processed the existing RNA expression data to generate expression profiles for a total of 19,192 genes (see Methods).

In the original study dataset, associations between gene expression and weight change were controlled for sex and race. Weight/height traits were frequently related to demographic characteristics [18, 23]. To more accurately control for age, sex, and race as correlated to weight and height traits, we computed the association levels between each of the demographic and clinical variables (Table 3). Using this method, when an association between demographic and clinical variables was noted, it was controlled for demographic characteristics. We found that weight change variables were not associated with any demographic variable, and age was not associated with any weight and height trait in this study. Height, weight, and BMI variables showed significant associations with sex at all time points (baseline, and 3, 6, and 12 months) and with race at baseline. Therefore, when we calculated the association between eigenvectors and clinical variables, we adjusted height/weight traits for sex and race at baseline and for sex at other time points.

**Table 3:** Assessment of associations between clinical and demographic characteristics

Clinical characteristics	Association (*p*-value)		
	Age at Transplantation (by linear regression)	Gender (by Analysis of Variance)	Race (by Analysis of Variance)
Height	0.542	1.22 × 10^−5^	0.282
Weight, baseline	0.737	4.59 × 10^−6^	0.049
Weight at 3 months	0.672	3.25 × 10^−5^	0.066
Weight at 6 months	0.939	6.66 × 10^−6^	0.088
Weight at 12 months	0.991	7.38 × 10^−5^	0.216
BMI at baseline	0.235	2.33 × 10^−3^	0.037
BMI at 3 months	0.242	0.013	0.074
BMI at 6 months	0.643	3.36 × 10^−3^	0.121
BMI at 12 months	0.697	0.011	0.429
Weight change percentage at 3 months	0.739	0.928	0.849
Weight change percentage at 6 months	0.276	0.778	0.542
Weight change percentage at 12 months	0.476	0.442	0.292

We further assessed whether gene expression data could be affected by confounding factors at baseline. To this end, we computed the first three principal components of gene expression data and related them to demographic characteristics (gender, age group and race), clinical parameters (glucose and creatinine), and preexisting diseases (hyperlipidemia, hypertension, diabetes and depression). Ages were divided into binary groups by age (above or below age 50). Age was divided into two even groups (above or below age 50) in order to better compare age with sex and race (both categorical variables). Additionally, age was tested as a continuous variable and as a continuous variable age was not significantly correlated with the first three principal components of microarray data. For each discrete variable, an ANOVA model was fit between the first three principal components and the variable. For each quantitative variable, a linear regression model was fit between the first three principal components and the variable. The *p*-values for all variables were retrieved and adjusted for multiple testing according to the Benjami-Hochberg procedure [21]. The outcome (Supplementary Table S1) shows that none of these variables is significantly associated with the first three principal components of the gene expression data. This analysis suggests that potential confounding factors are not substantially affecting gene expression analysis.

### Gene co-expression networks and their relationship to obesity traits and fat-related traits in kidney transplant patients

**Fig. 1:**
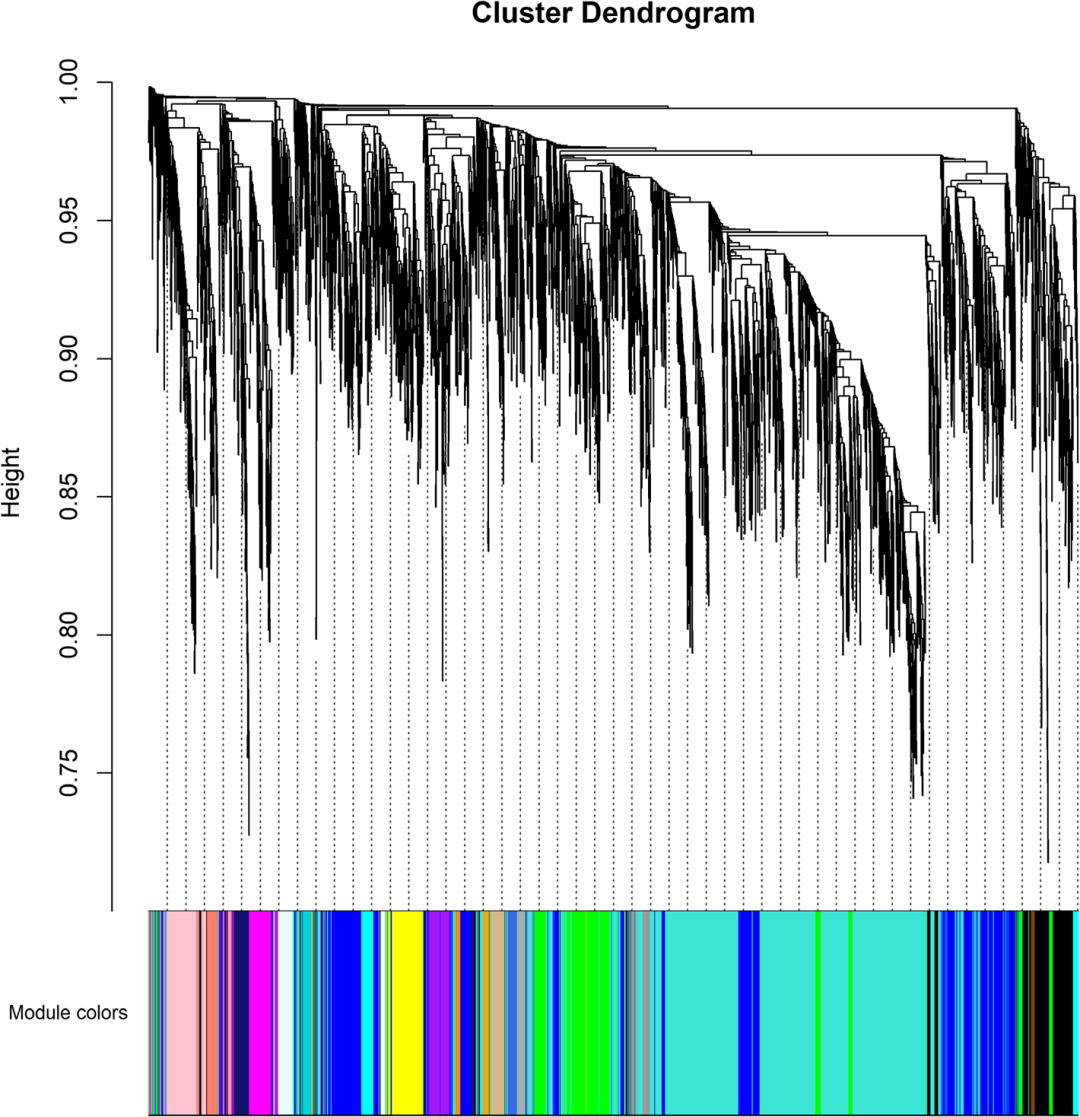
Dendrogram showing the module-gene relationships generated by the WGCNA software

**Fig. 2:**
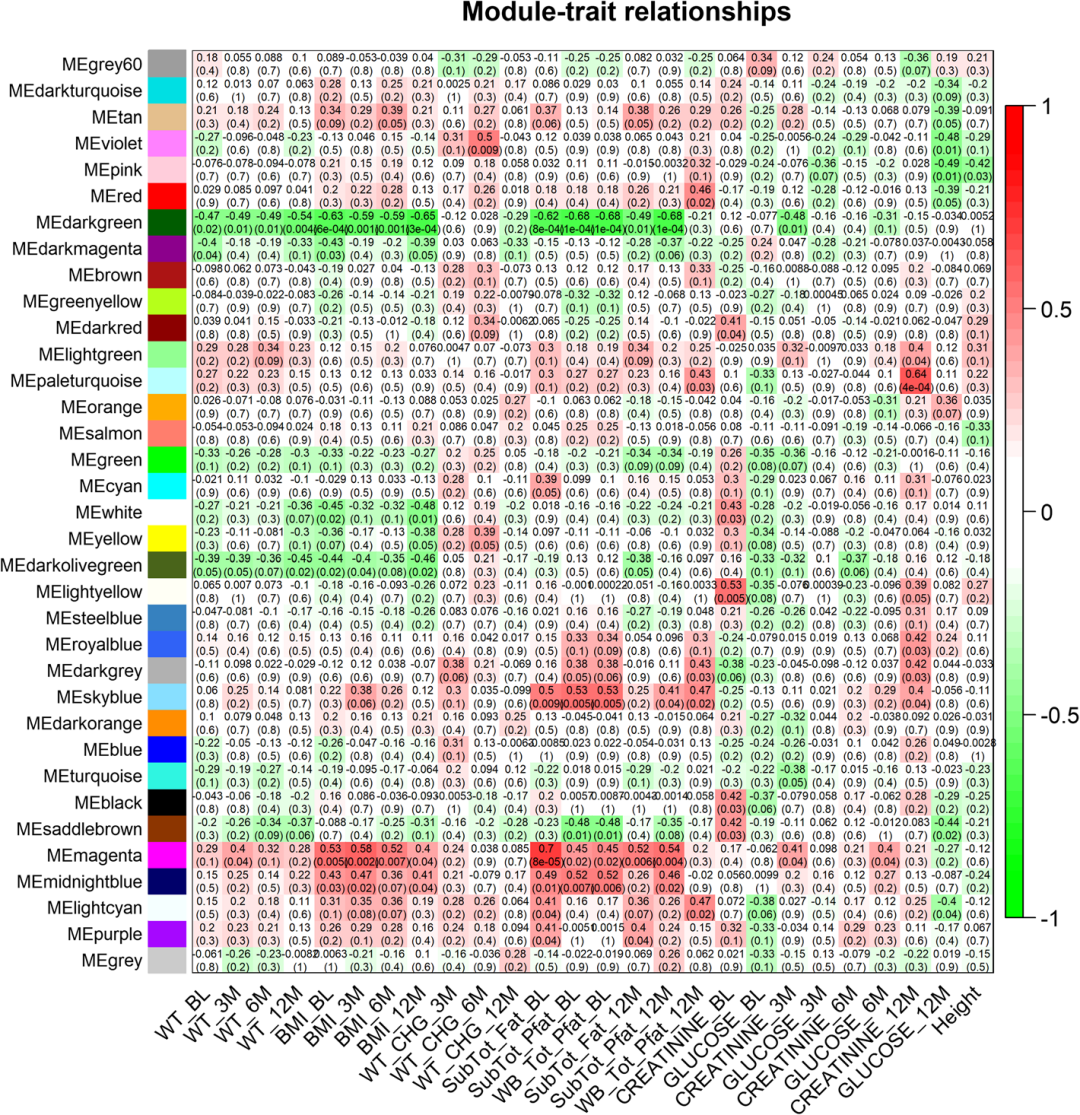
Detection of gene modules associated with clinical traits. Heatmap plot illustrating module-trait relationships. Each element in the heatmap contains a correlation value based on the correlation between gene modules (y-axis) and corresponding clinical traits and *p*-values (X-axis). The strength of the correlation is depicted by its color

Gene co-expression networks can reveal coordinative behaviors of gene transcription for diseases or medical interventions. We conducted Weighted Gene Correaltion Network Analysis (WGCNA) to obtain a better understanding of gene expression mechanisms underlying obesity-related traits. 5758 (30%) of the most highly expressed genes were examined to reduce the noise (i.e., genes not actually expressed). Fitting of scale-free topology identified 35 gene modules with sizes ranging from 10 to 1451 genes and a median size of 49 (Fig. 1). We then examined associations between these 35 gene modules and 26 clinical variables (see Methods, Fig. 2). In order to ensure detection of reliable associations, the *p*-values obtained from the WGCNA software were further adjusted for multiple testing according to the Benjami-Hochberg approach. At an adjusted *p*-value cutoff of 0.05, two modules showed association with at least one clinical trait: the “magenta” module was correlated with SubTot_Fat_BL; the “darkgreen” module was correlated with SubTot_Pfat_BL and WB_Tot_Pfat_BL.

**Fig. 3:**
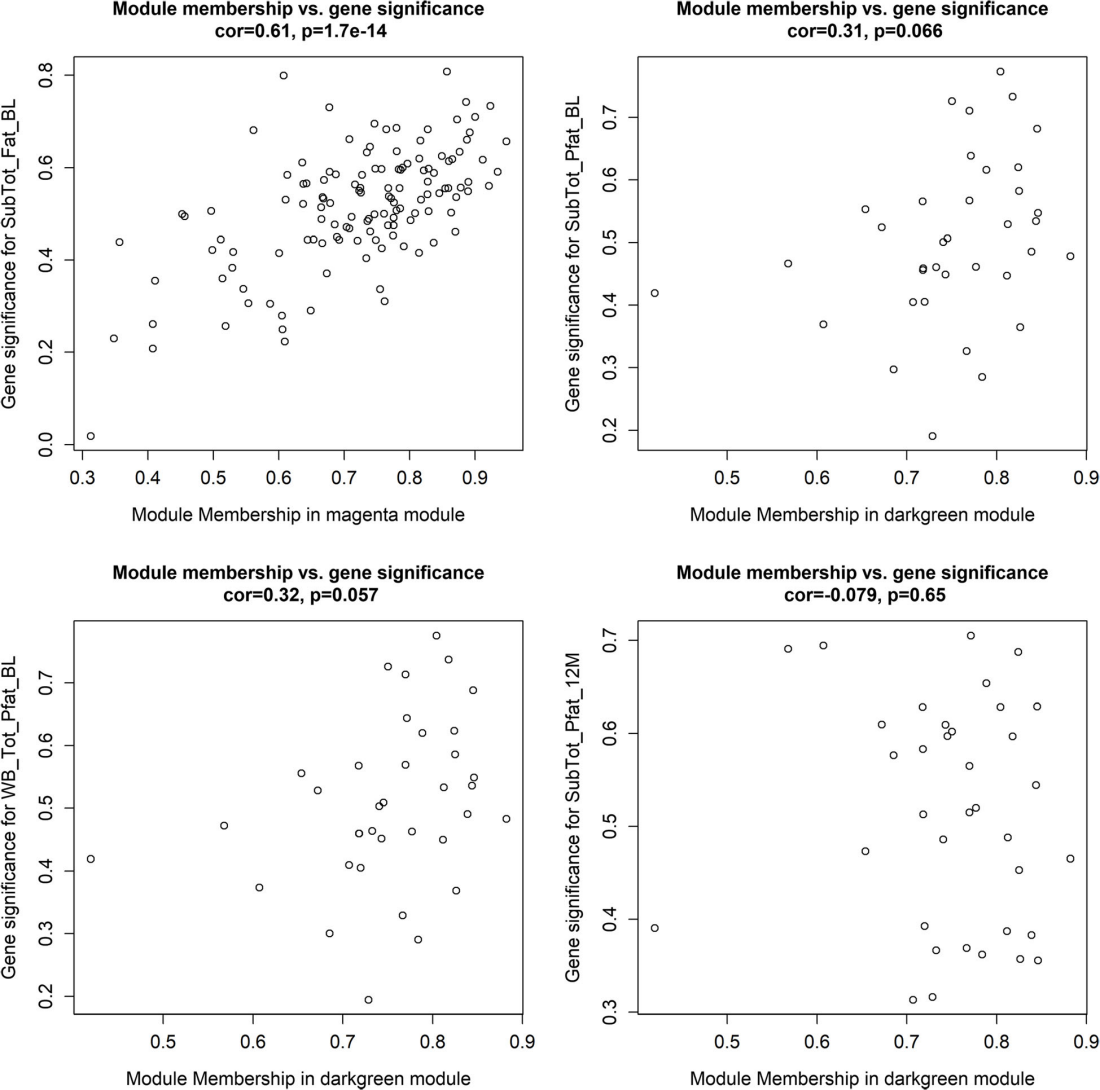
Correlations between gene module memberships and gene significance for the modules associated with clinical traits

For each module, the eigengene represents its major gene expression pattern among its component gene members. The gene module membership measures how close a gene’s expression profile is to its eigengene, and it is expected that genes with higher module memberships are more functionally important in the module and are more likely to be correlated with clinical traits. In turn, if genes in a module show high correlations between module memberships and gene significance (for association with traits), that suggests that the association between the gene module and the clinical trait is highly reliable. To this end, we made plot gene module memberships and gene significance for the modules associated with clinical traits (Fig. 3). The magenta module showed a highly significant module-membership to gene-significance correlation with the SubTot_Fat_BL trait. The darkgreen module held moderate module-membership to gene-significance correlations with SubTot_Pfat_BL and WB_Pfat_BL. This analysis confirmed that both the magenta and darkgreen modules are functionally important for obesity-related traits.

### Functional landscapes of obesity-related gene modules

We then utilized GO enrichment analysis to examine the association between functional properties and clinical traits within the magenta and darkgreen modules. Both modules showed GO enrichment at an FDR-corrected *p*-value of 0.05. The magenta module contains 129 genes. The GO term enrichment for the co-expression modules is shown in supplementary Table S2. GO enrichment of the magenta module identified multiple biological components and processes associated with SubTot_Fat_BL, including immune system processes and lipid metabolism. Enrichment of the darkgreen module using GOStat found an association of SubTot_Pfat_BL and WB_Tot_Pfat_BL with heart and vasculature development.

To further explore the functionality of the magenta and darkgreen module, we classified the top 10% of genes with the highest connectivity as hub genes. The top 2 hub genes within the magenta module were *ITGAM* (92 connections), *CD68* (89 connections). The top 2 hub genes within the darkgreen module were *CRLS1* (18 connections) and *ACSS3* (12 connections).

**Fig. 4:**
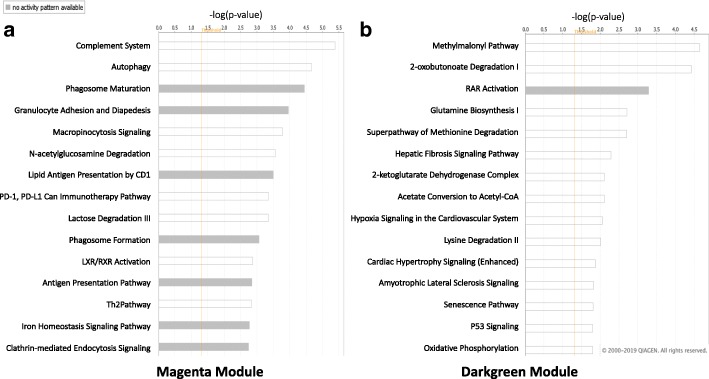
Top canonical pathways for magenta and darkgreen modules generated by IPA. **a** Magenta canonical pathway. **b** Darkgreen canonical pathway

IPA-identified canonical pathways and gene networks within the magenta and darkgreen module. Canonical pathways within the magenta module included: phagosome maturation, autophagy, lipid antigent presentation, and inflammatory signaling (e.g. Il-6 and Il-8) (Fig. 4). Eight networks where identified in the magenta module; Top networks were associated with immune cell trafficking (score 12), cardiovascular disease (score 18), lipid metabolism (score 22), and inflammatory response (score 11). Canonical pathways within the darkgreen module included: hypoxia signaling in the cardiovascular system, cardiac hypertrophy signaling, and the Methylmalonyl Pathway. Top networks in the darkgreen module were associated with infectious diseases (score 30), gastrointestinal disease (score 27), and cardiovascular system development and function (score 22).

## Discussion

This study sought to identify sets of genes that could provide insight into the genetic underpinnings of obesity-related traits in kidney transplant recipients. Using gene expression data from adipose tissue of kidney transplant recipients, we created gene co-expression networks and correlated them to clinical variables. Previous work has identified individual genes associated with weight gain in kidney transplant populations [18, 23], but to our knowledge no studies have incorporated WGCNA in similar cohorts. Using WGCNA, we found two modules, the magenta module and the darkgreen module, to be significantly correlated with obesity-related traits at baseline.

GO enrichment analysis and identification of hub genes revealed plausible biological functions of genes within the magenta and darkgreen modules. GO enrichment analysis identified that among the 129 genes comprising the magenta model, multiple genes are involved in immune function, inflammation, lipid metabolism, and cardiovascular disease. This is supported by the role of the hub genes within the magenta module. As mentioned previously, the top two hub genes in the magenta module are *ITGAM* and *CD68. ITGAM* plays a role in the adhesion of monocytes, macrophages, and granulocytes and the uptake of pathogens [24, 25]. *CD68* codes for a glycoprotein expressed in monocytes and macrophages and is related to the innate immune system and low-density lipoprotein (LDL) oxidation in Atherogenesis [26, 27]. Thus, GO enrichment and hub genes are consistent, both suggesting the magenta module may hold immune-related, metabolic, and cardiovascular roles in kidney transplant patients.

The biological processes identified by GO enrichment and the hub genes’ function are also consistent with our network analysis using IPA. Network analysis of the magenta module revealed that 15 genes within this module, including *ITGAM* and *CD68*, have immune-related functions. Additionally, within the magenta module, IPA identified 2 networks associated with lipid metabolism (22 genes) and metabolic disease (11 genes). Other genes within the magenta module play a role in metabolic functions and obesity including genes that code for lipase (*LIPA)*, phospholipid transfer protein (*PTLP),* [28] and lipid metabolism (e.g., *LPIN1)* [29]. This suggests that there is a relationship between metabolism, immune response, inflammatory-related genes, and body-fat in kidney transplant patients.

Like the magenta module, the darkgreen module contains genes associated with lipid metabolism and cardiovascular processes. The darkgreen module was enriched for cardiovascular processes including heart and vasculature development. Additionally, the darkgreen module contains genes associated with cellular energy expenditure (e.g. mitochondrion and sodium: potassium-exchanging ATPase activity). The top 2 hubgenes, *CRLS1* and *ACSS3* are involved in lipid metabolism, including the catalysis of key phospholipids and acetyl-CoA from short-chain fatty acids [30, 31]. Network analysis of the darkgreen module was also consistent with the GO enrichment analysis and the function of the hub genes, containing three networks associated with the immune response (e.g. infectious diseases), and cardiovascular processes (e.g. cardiovascular system development and function). Thus, body-fat associated genes within the darkgreen module are associated with cardiovascular health, lipid metabolism, and cellular energy expenditure. Both modules from our analysis are tied to immunological responses such as inflammation, suggesting the immune system may play a role in regulating obesity-related traits (e.g. total subcutaneous body fat) in kidney transplant patients.

The findings of the current study are supported by previous studies which have found immunological, inflammatory, and metabolic genetic drivers are influential in the determination of obesity [32–34]. Only one previous study by Muniandy et al. used adipose tissue (subcutaneous) to study clinical changes associated with obesity; The study found that the heavier twin in BMI-discordant monozygotic twin pairs displayed an upregulation of inflammation and other immunological pathways/genes (e.g. *IFI30* and *CCL18)* [32]. Other studies have used blood samples to examine differential gene expression and weighted gene co-expression network analysis from a cohort of monozygotic twins [27]. A study by Wang et al. found 32 differentially expressed genes (DEGs) that were upregulated in the higher BMI twins including DEGs associated with obesity (e.g. *NAMPT*, *TLR9*, *PTGS2*, *HBD*, and *PCSK1N*), immunological (e.g., *TLR9*), and metabolic functions (e.g., *PTGS2*). Wang et al. also used WGCN to identify two modules strongly correlated to BMI including genes responsible for regulation of phospholipase activity, high density lipoprotein particle clearance, and voltage-gated potassium channel complex. Animal studies have also found that an association between obesity-related genes and inflammation. Studies have found inflammatory signaling affects adipocyte insulin receptors and causes insulin resistance, which further contributed to fatty acid accumulation and obesity [35, 36].

Gene modules were constructed based on gene expression at baseline. As these modules were constructed at baseline, we did not observe significant associations between gene modules and clinical traits beyond the baseline (i.e. following kidney transplant). As presented by Cashion et al. additional variables (aside from gene associations) contribute to clinical traits following transplantation [19]. For example younger age, higher carbohydrate consumption, higher trunk fat, and higher perception of mental health quality of life are predictors of weight gain following kidney transplantation [19].

Importantly, our analysis is limited to the clinical and demographic variables collected in the parent study. Additionally, only a portion of the participants in the parent study signed the repository consent and had available microarray data. Future studies should examine the co-expression patterns in larger more diverse populations of kidney transplant patients. Studies must also examine whether the obesity-related gene co-expression patterns observed in this study apply to weight gain in the general population.

## Conclusion

The current study examined gene co-expression patterns associated with obesity-related traits in kidney transplant recipients. Utilizing WGCNA, we generated gene co-expression networks associated with obesity-related traits to highlight possible gene modules responsible for obesity, and assessed these modules’ pathways, molecular functions, and gene-gene interactions with obesity-related traits. A total of 35 co-expression modules were detected, two modules were associated with clinical traits. These modules are involved in metabolic and immune processes (including genes involved in lipid metabolism and immune-related functions) and are associated with multiple obesity-related traits, total subcutaneous body fat and whole-body fat percent. This study offers a deeper understanding of the gene network properties underlying obesity-related traits and provides new insights regarding the biological processes underlying obesity in kidney transplant patients.

## Supplementary information

**Additional file 1: Fig. S1.** Scale-Free Topology Model Fit.

**Additional file 2: Table S1.** FDR-adjusted *p*-values of ANOVA/linear regression modules between the first three components of gene expression data and demographic/disease characteristics at baseline. **Table S2.** Enriched GO terms in the clinical trait-associated modules.

## Data Availability

Microarry data is available at the National Center for Biotechnology Information’s Gene Exression Omnibus (GEO) Database at accession number GSE33070 https://www.ncbi.nlm.nih.gov/geo/query/acc.cgi?acc=GSE33070. Additional data is available upon request from senior author paule.joseph@nih.gov.

## Abbreviations

BMI_BL: Body mass index at base line
CKD: Chronic Kidney Disease
CREATININE_12M: creatinine 12 months following transplantation
CREATININE_3M: creatinine 3 months following transplantation
CREATININE_6M: creatinine 6 months following transplantation
CREATININE_BL: creatinine at baseline
DEXA: Dual-energy X-ray absorptiometry
ESRD: End Stage Renal Disease
GLUCOSE_12M: Glucose 3 months following transplantation
GLUCOSE_3M: Glucose 3 months following transplantation
GLUCOSE_6M: Glucose 3 months following transplantation
GLUCOSE_BL: Glucose at baseline
GO: Gene Ontology
GWAS: Genome Wide Association Studies
SubTot_Fat_BL: Subcutaneous body fat at baseline
SubTot_Pfat_BL: Percent of subcutaneous body fat at baseline
WB_Tot_Pfat_BL: Whole-body fat percent at baseline
WGCNA: Weighted Gene Correlation Network Analysis
WT_BL: Weight at baseline
