# Supplementary figures and images for: Gene co-expression networks are associated with obesity-related traits in kidney transplant recipients
Source: BMC Med Genomics. 2020 Mar 10;13:37. doi: 10.1186/s12920-020-0702-5 (PMC7063809; doi:10.1186/s12920-020-0702-5)

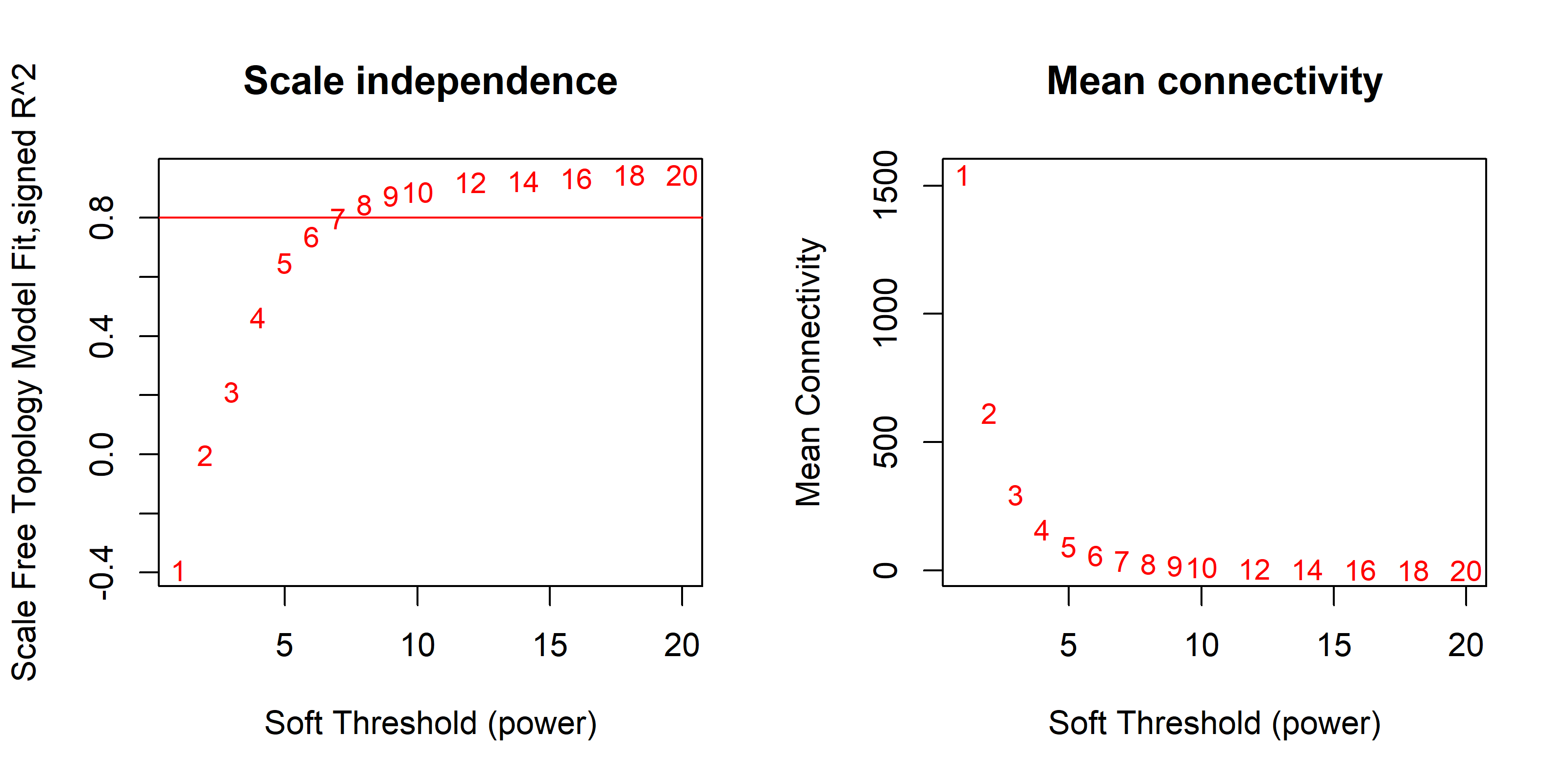

Supplement: Supplementary file 1 — Additional file 1: Fig. S1. Scale-Free Topology Model Fit. [file 12920_2020_702_MOESM1_ESM.png]
