# Supplementary material for: Gene co-expression networks are associated with obesity-related traits in kidney transplant recipients
Source: BMC Med Genomics. 2020 Mar 10;13:37. doi: 10.1186/s12920-020-0702-5 (PMC7063809; doi:10.1186/s12920-020-0702-5)
Supplement: Supplementary file 2 — Additional file 2: Table S1. FDR-adjusted p-values of ANOVA/linear regression modules between the first three components of gene expression data and demographic/disease characteristics at baseline. Table S2. Enriched GO terms in the clinical trait-associated modules. [file 12920_2020_702_MOESM2_ESM.docx]

**Table S1. FDR-adjusted *p*-values of ANOVA/linear regression modules between the first three components of gene expression data and demographic/disease characteristics at baseline**

| **Principal component** | **Demographic characteristics** | | | **Clinical Values** | | **Pre-existing diseases** | | | |
| --- | --- | --- | --- | --- | --- | --- | --- | --- | --- |
|  | Sex | Age group^1^ | Race | Creatinine | Glucose | Hyperlipidemia | Hypertension | Diabetes | Depression |
| PC1 | 0.967 | 0.649 | 0.967 | 0.967 | 0.649 | 0.649 | 0.981 | 0.981 | 0.967 |
| PC2 | 0.990 | 0.784 | 0.967 | 0.649 | 0.990 | 0.649 | 0.649 | 0.981 | 0.981 |
| PC3 | 0.649 | 0.649 | 0.649 | 0.514 | 0.649 | 0.410 | 0.981 | 0.649 | 0.967 |

^1^Ages were divided into two groups by age of 50

**Table S2. Enriched GO terms in the clinical trait-associated modules**

| **Module** | **Size** | **Associated clinical traits** | **Enriched GO terms** | **Count** | **Total** | **FDR-**  **adjusted**  ***p*-value** |
| --- | --- | --- | --- | --- | --- | --- |
| Magenta | 129 | SubTot_Fat_BL | GO:0005773: (C) vacuole | 22 | 102 | 2.08×10^-12^ |
|  |  |  | GO:0005764: (C) lysosome | 20 | 91 | 1.08×10^-11^ |
|  |  |  | GO:0000323: (C) lytic vacuole | 20 | 91 | 1.08×10^-11^ |
|  |  |  | GO:0004872: (M) signaling receptor activity | 20 | 255 | 1.79×10^-5^ |
|  |  |  | GO:0044459: (C) obsolete plasma membrane part | 28 | 438 | 2.45×10^-5^ |
|  |  |  | GO:0006954: (P) inflammatory response | 12 | 73 | 3.25×10^-5^ |
|  |  |  | GO:0005615: (C) extracellular space | 13 | 92 | 5.24×10^-5^ |
|  |  |  | GO:0009611: (P) response to wounding | 14 | 113 | 8.30×10^-5^ |
|  |  |  | GO:0005887: (C) integral component of plasma membrane | 20 | 283 | 1.11×10^-4^ |
|  |  |  | GO:0031226: (C) intrinsic component of plasma membrane | 20 | 283 | 1.11×10^-4^ |
|  |  |  | GO:0006952: (P) defense response | 13 | 115 | 4.11×10^-4^ |
|  |  |  | GO:0009605: (P) response to external stimulus | 15 | 159 | 6.00×10^-4^ |
|  |  |  | GO:0005886: (C) plasma membrane | 34 | 673 | 6.35×10^-4^ |
|  |  |  | GO:0002376: (P) immune system process | 16 | 191 | 1.13×10^-3^ |
|  |  |  | GO:0005044: (F) scavenger receptor activity | 4 | 8 | 1.49×10^-3^ |
|  |  |  | GO:0016021: (C) integral component of membrane | 47 | 1115 | 2.56×10^-3^ |
|  |  |  | GO:0031224: (C) intrinsic component of membrane | 47 | 1115 | 2.56×10^-3^ |
|  |  |  | GO:0042611: (C) MHC protein complex | 4 | 10 | 3.52×10^-3^ |
|  |  |  | GO:0006955: (P) immune response; | 12 | 131 | 4.11×10^-3^ |
|  |  |  | GO:0004553: (M) hydrolase activity, hydrolyzing O-glycosyl compounds | 6 | 33 | 6.14×10^-3^ |
|  |  |  | GO:0044421: (C) obsolete extracellular region part | 14 | 183 | 6.91×10^-3^ |
|  |  |  | GO:0044425: (C) membrane part | 53 | 1376 | 8.94×10^-3^ |
|  |  |  | GO:0042613: (C) MHC class II protein complex | 3 | 6 | 0.0104 |
|  |  |  | GO:0004871: (F) obsolete signal transducer activity | 22 | 419 | 0.0104 |
|  |  |  | GO:0060089: (M) molecular transducer activity | 22 | 419 | 0.0104 |
|  |  |  | GO:0042592: (P) homeostatic process | 10 | 111 | 0.0132 |
|  |  |  | GO:0002504: (P) antigen processing and presentation of peptide or polysaccharide antigen via MHC class II | 3 | 7 | 0.0158 |
|  |  |  | GO:0019882: (P) antigen processing and presentation | 4 | 16 | 0.0166 |
|  |  |  | GO:0055080: (P) cation homeostasis | 6 | 43 | 0.0173 |
|  |  |  | GO:0015925: (M) galactosidase activity | 2 | 2 | 0.0173 |
|  |  |  | GO:0004565: (M) beta-galactosidase activity | 2 | 2 | 0.0173 |
|  |  |  | GO:0019725: (P) cellular homeostasis | 8 | 78 | 0.0179 |
|  |  |  | GO:0006958: (P) complement activation, classical pathway | 3 | 8 | 0.0197 |
|  |  |  | GO:0002455: (P) humoral immune response mediated by circulating immunoglobulin | 3 | 8 | 0.0197 |
|  |  |  | GO:0004197: (M) cysteine-type endopeptidase activity | 5 | 31 | 0.0225 |
|  |  |  | GO:0051179: (P) localization | 39 | 970 | 0.0240 |
|  |  |  | GO:0006935: (P) chemotaxis | 5 | 32 | 0.0240 |
|  |  |  | GO:0042330: (P) taxis | 5 | 32 | 0.0240 |
|  |  |  | GO:0002526: (P) cute inflammatory response | 4 | 19 | 0.0240 |
|  |  |  | GO:0008021: (C) synaptic vesicle | 3 | 9 | 0.0251 |
|  |  |  | GO:0044437: (C) obsolete vacuolar part | 5 | 33 | 0.0252 |
|  |  |  | GO:0005774: (C) vacuolar membrane | 5 | 33 | 0.0252 |
|  |  |  | GO:0055066: (P) obsolete di-, tri-valent inorganic cation homeostasis | 5 | 33 | 0.0252 |
|  |  |  | GO:0051234: (P) establishment of localization | 35 | 847 | 0.0252 |
|  |  |  | GO:0006956: (P) complement activation | 3 | 10 | 0.0300 |
|  |  |  | GO:0002541: (P) activation of plasma proteins involved in acute inflammatory response | 3 | 10 | 0.0300 |
|  |  |  | GO:0006810: (P) transport | 34 | 825 | 0.0300 |
|  |  |  | GO:0007610: (P) behavior | 7 | 70 | 0.0300 |
|  |  |  | GO:0006907: (P) pinocytosis | 2 | 3 | 0.0309 |
|  |  |  | GO:0051181: (P) cofactor transport | 2 | 3 | 0.0309 |
|  |  |  | GO:0008329: (M) pattern recognition receptor activity | 2 | 3 | 0.0309 |
|  |  |  | GO:0016668: (M) oxidoreductase activity, acting on a sulfur group of donors, NAD(P) as acceptor | 2 | 3 | 0.0309 |
|  |  |  | GO:0004888: (M) transmembrane signaling receptor activity | 10 | 135 | 0.0309 |
|  |  |  | GO:0005125: (M) cytokine activity | 5 | 38 | 0.0377 |
|  |  |  | GO:0006811: (P) ion transport | 11 | 164 | 0.0380 |
|  |  |  | GO:0016023: (C) cytoplasmic membrane-bound vesicle | 10 | 141 | 0.0392 |
|  |  |  | GO:0019724: (P) B cell mediated immunity | 3 | 12 | 0.0423 |
|  |  |  | GO:0016064: (P) immunoglobulin mediated immune response | 3 | 12 | 0.0423 |
|  |  |  | GO:0031988: (C) membrane-bound vesicle | 10 | 144 | 0.0432 |
|  |  |  | GO:0050801: (P) ion homeostasis | 6 | 58 | 0.0432 |
|  |  |  | GO:0005031: (M) tumor necrosis factor receptor activity | 2 | 4 | 0.0491 |
|  |  |  | GO:0051180: (P) vitamin transport | 2 | 4 | 0.0491 |
|  |  |  | GO:0005035: (M) death receptor activity | 2 | 4 | 0.0491 |
|  |  |  | GO:0006817: (P) phosphate transport | 4 | 26 | 0.0491 |
|  |  |  | GO:0030003: (P) cellular cation homeostasis | 5 | 42 | 0.0491 |
|  |  |  | GO:0031410: (C) cytoplasmic vesicle | 11 | 173 | 0.0494 |
| Darkgreen | 36 | SubTot_Pfat_BL  WB_Tot_Pfat_BL | [GO:0014706](http://amigo.geneontology.org/cgi-bin/amigo/go.cgi?view=details&search_constraint=terms&query=GO:0014706): (P) striated muscle tissue development | 4 | 31 | 0.0136 |
|  |  |  | [GO:0007507](http://amigo.geneontology.org/cgi-bin/amigo/go.cgi?view=details&search_constraint=terms&query=GO:0007507): (P) heart development | 4 | 31 | 0.0136 |
|  |  |  | [GO:0005739](http://amigo.geneontology.org/cgi-bin/amigo/go.cgi?view=details&search_constraint=terms&query=GO:0005739): (C) mitochondrion | 10 | 367 | 0.0136 |
|  |  |  | [GO:0005890](http://amigo.geneontology.org/cgi-bin/amigo/go.cgi?view=details&search_constraint=terms&query=GO:0005890): (C) sodium: potassium-exchanging ATPase complex | 2 | 3 | 0.0136 |
|  |  |  | [GO:0048738](http://amigo.geneontology.org/cgi-bin/amigo/go.cgi?view=details&search_constraint=terms&query=GO:0048738): (P) cardiac muscle tissue development | 2 | 3 | 0.0136 |
|  |  |  | [GO:0005391](http://amigo.geneontology.org/cgi-bin/amigo/go.cgi?view=details&search_constraint=terms&query=GO:0005391): (M) sodium: potassium-exchanging ATPase activity | 2 | 3 | 0.0136 |
|  |  |  | [GO:0048514](http://amigo.geneontology.org/cgi-bin/amigo/go.cgi?view=details&search_constraint=terms&query=GO:0048514): (P) blood vessel morphogenesis | 4 | 52 | 0.0306 |
|  |  |  | [GO:0007517](http://amigo.geneontology.org/cgi-bin/amigo/go.cgi?view=details&search_constraint=terms&query=GO:0007517): (P) muscle organ development | 4 | 56 | 0.0352 |
|  |  |  | [GO:0016421](http://amigo.geneontology.org/cgi-bin/amigo/go.cgi?view=details&search_constraint=terms&query=GO:0016421): (M) CoA carboxylase activity | 2 | 6 | 0.0352 |
|  |  |  | [GO:0001570](http://amigo.geneontology.org/cgi-bin/amigo/go.cgi?view=details&search_constraint=terms&query=GO:0001570): (P) vasculogenesis | 2 | 6 | 0.0352 |
|  |  |  | [GO:0001568](http://amigo.geneontology.org/cgi-bin/amigo/go.cgi?view=details&search_constraint=terms&query=GO:0001568): (P) blood vessel development | 4 | 62 | 0.0352 |
|  |  |  | [GO:0001944](http://amigo.geneontology.org/cgi-bin/amigo/go.cgi?view=details&search_constraint=terms&query=GO:0001944): (P) vasculature development | 4 | 62 | 0.0352 |
|  |  |  | [GO:0044429](http://amigo.geneontology.org/cgi-bin/amigo/go.cgi?view=details&search_constraint=terms&query=GO:0044429): (C) obsolete mitochondrial part | 7 | 241 | 0.0372 |
|  |  |  | [GO:0016885](http://amigo.geneontology.org/cgi-bin/amigo/go.cgi?view=details&search_constraint=terms&query=GO:0016885): (M) ligase activity, forming carbon-carbon bonds | 2 | 7 | 0.0378 |
|  |  |  | [GO:0050662](http://amigo.geneontology.org/cgi-bin/amigo/go.cgi?view=details&search_constraint=terms&query=GO:0050662): (M) coenzyme binding | 4 | 67 | 0.0378 |
